# Global Profiling of Dynamic Alternative Splicing Modulation in Arabidopsis Root upon *Ralstonia solanacearum* Infection

**DOI:** 10.3390/genes11091078

**Published:** 2020-09-15

**Authors:** Ning Qin, Ruize Zhang, Mancang Zhang, Yang Niu, Shouyang Fu, Yisa Wang, Dongdong Wang, Yue Chen, Cuizhu Zhao, Qin Chen, Haibin Lu

**Affiliations:** 1 State Key Laboratory of Crop Stress Biology for Arid Areas, College of Agronomy, Northwest A&F University, Yangling 712100, China; qn721@nwafu.edu.cn (N.Q.); zhangruize@nwafu.edu.cn (R.Z.); 13569679584@163.com (M.Z.); 18238791002@163.com (Y.N.); 18438616378@163.com (S.F.); wys17337764859@163.com (Y.W.); dongdong-1025@hotmail.com (D.W.); xnchenyue@nwafu.edu.cn (Y.C.); zhaocuizhu2002@163.com (C.Z.); 2 College of Food Science and Engineering, Northwest A&F University, Yangling 712100, China

**Keywords:** alternative splicing, *Ralstonia solanacearum*, *Arabidopsis thaliana*, plant defense, splicing factors/RNA-binding proteins

## Abstract

Alternative splicing (AS) is an important mechanism by which eukaryotes regulate transcription and protein diversity. The dynamic changes in AS that occur on a genome-wide scale during interactions between plant roots and pathogens remain unknown. Here, we used the interaction between Arabidopsis and *Ralstonia solanacearum* as a model to explore the AS changes that take place during the response of roots to infection by means of high-throughput RNA-sequencing. We showed that dynamic changes in AS occur much earlier than changes at the level of transcription during *R.* *solanacearum* infection. Comparing genes that are regulated at the transcriptional and AS levels indicated that there are few common genes between differentially spliced genes (DSGs) and differentially expressed genes (DEGs). The functional gene ontology (GO) analysis identified that the enriched GO terms for the DSGs were different from those of the DEGs. The DSGs were over-represented in GO terms associated with post-transcriptional and translational regulations, suggesting that AS may act on RNA stability and during post-translation, thus affecting the output of plant defense molecules. Meanwhile, changes in DSGs were infection stage-specific. Furthermore, the nucleotide binding domain and leucine-rich repeat proteins and receptor-like kinases, key regulators in plant immunity, were shown to undergo dynamic changes in AS in response to *R. solanacearum*. Taken together, AS, along with transcription, modulates plant root defense to *R. solanacearum* through transcriptome reprogramming.

## 1. Introduction

As plants have a sessile lifestyle, they are vulnerable to adverse conditions in their environment that impact their growth and development. In particular, *Ralstonia solanacearum* is a biotrophic soil-borne phytopathogen that causes bacterial wilt disease on many economically important crops, such as tobacco, potato and tomato [1]. The pathogen invades plants through natural openings or wound sites on the roots and crosses the cortex and endodermis before reaching the root xylem, where it begins to colonize. The bacteria then spread to the aerial parts of the plant and cause wilting symptoms [2]. In addition, due to its soil-born nature, long persistence, wide host range, broad geographical distribution and the lack of an efficient means to control it, *R. solanacearum* is considered to be one of most destructive plant pathogenic bacteria [1].

Innate immunity in plants relies on two major layers of defense [3]. The first tier involves pathogen-associated molecular pattern (PAMPs)-triggered immunity (PTI), which is activated by the recognition of PAMPs in microbes through pattern recognition receptors (PRRs), including receptor-like proteins (RLPs) and receptor-like kinases (RLKs) located on the plant cell surface. The second tier comprises effector-triggered immunity (ETI), through which plants utilize intracellular disease resistance (R) proteins to recognize pathogen effectors and activate the defense response. In general, ETI is often accompanied by the hypersensitive response, whereby local cell death occurs in the infected plant tissue. Host-pathogen interactions between *R. solanacearum* and the model plant *Arabidopsis thaliana* have been established over 20 years of research [4]. However, apart from evidence of the R proteins RRS1-R and RPS4, the mechanism that underlies plant defense to *R. solanacearum* is not well-understood [5]. In addition, phytohormones such as salicylic acid, ethylene, abscisic acid and jasmonate are partly involved in plant susceptibility or resistance to *R. solanacearum* [6,7,8,9].

Alternative splicing (AS) is an important method of post-transcriptional regulation for gene expression. It generates different mRNA isoforms from the same precursor mRNA (pre-mRNA) in eukaryotes and increases proteome diversity by generating protein variants with different functions [10]. AS can also generate mRNA isoforms containing a premature stop codon. Many of these isoforms are likely to be targeted and degraded by nonsense-mediated decay, thus affecting RNA stability [11]. The outcome of AS is affected by the concerted action of splicing factors (SFs) and RNA-binding proteins (RBPs), which directly interact with cis-regulatory elements in the pre-mRNA. In humans, a genome-wide analysis has shown that more than 95% of the intron-containing genes are alternatively spliced [12] and about 15% of genetic diseases are caused by mutations affecting the splicing mechanism [13]. Extensive studies on AS have been undertaken in various plants, at different developmental stages, in an array of tissues [14,15] and during plant responses to biotic or abiotic stresses, such as cold [16] and *Pseudomonas syringae* infection [17]. AS represents a newly identified mechanism of regulating plant defense [18,19]. The process has been shown to affect plant defense against pathogens by producing isoforms of plant resistance genes, including nucleotide-binding site-leucine-rich repeat (NBS-LRR) genes, PRR and phytohormonal signaling genes [20]. Moreover, the expression levels, localization and activities of SFs and RBPs under different conditions determine the selection of splicing sites and affect the AS output [21,22]. When trans-acting factors are mis-expressed, the host’s resistance to pathogens is altered [20,23].

High-throughput transcriptome sequencing provides new insight into the analysis of AS regulation on a genome-wide scale. Previous genome-wide microarray and RNA-sequencing (RNA-seq) studies have been focused mainly at the transcriptional level in Arabidopsis affected by *R. solanacearum*. Thousands of differentially expressed genes (DEGs) have been reported at the early stage of infection [9,24]. However, the extent and dynamics of AS in plant root responses to soil-borne phytopathogens is unknown. In this study, we exploited the high temporal resolution of RNA-seq to explore the dynamic changes that take place in AS chronologically in Arabidopsis roots following *R. solanacearum* infection. We found rapid and dynamic changes in AS following infection by this pathogen. Hundreds of genes exhibited differential AS events (DSEs), while only a few displayed differential expressions at six h post inoculation (hpi). We also identified the AS events that occurred in some R proteins, receptor-like kinases, key phytohormonal signaling genes and SF/RBPs. Our data indicate that AS regulation, a post-transcriptional type of regulation mechanism, plays a critical role in plant resistance to *R. solanacearum* and provides a novel view of the complex regulation networks that are active during the interaction between *R. solanacearum* and Arabidopsis.

## 2. Materials and Methods

### 2.1. Plant Materials and R. solanacearum Infection

*Arabidopsis thaliana* Col-0 seed surfaces were sterilized with 0.02% TritonX-100 and 30% bleach and grown on Murashige Skoog (MS) without sucrose medium plates, which set vertically at 25 °C and 16-h light (9000 lux)/8-h dark conditions for 6 to 7 days. Six-to-seven-day-old Col-0 seedlings were inoculated with 10^7^ colony forming units (CFU) of *R. solanacearum* strain GMI1000 at 1 cm above the root apex and then grown in the same conditions aforementioned. Finally, root samples were collected at the 6 time points (0, 6, 12, 24, 48 and 72 h post-infection (hpi)).

### 2.2. RNA Extraction and Sequencing

The method of total RNA extraction was described in our previous study [9]. Electrophoresis was used to measure the degradation and contamination of total RNA. The RNA Nano 6000 Assay Kit of the Bioanalyzer 2100 system (Agilent Technologies, Santa Clara, CA, USA) was used to estimate the RNA integrity. Three micrograms of total RNA from each root sample was used for preparing the RNA-seq libraries. The manufacturer’s protocols (NEBNext^®^ UltraTM integrity RNA Library Prep kit for Illumina^®^) was used to construct sequencing libraries of 21 samples (the roots of *Arabidopsis thaliana* infected by *Ralstonia solanacearum* at 0, 6, 12, 24, 48 and 72 hpi, with three replications). The prepared libraries were sequenced on the Illumina Hi-seq platform and 150-bp paired-end reads were generated.

### 2.3. Data Quality Control and Read Mapping

Raw reads in the fastq format were firstly obtained through in-house perl scripts. Raw reads were filtered to generate clean reads by removing the adapter sequence, ploy-N and low-quality reads. An average of 33.9 million clean reads (ranging from 28.9 to 40.5) with Q30 > 90% were produced per sample. Above 94% of the clean reads were mapped on the TAIR10 reference genome downloaded from The Arabidopsis Information Resource (http://www.arabidopsis.org) using HISAT v2.0.4 with the default parameters (Table S1).

### 2.4. Analysis of Differential Alternative Splicing Events and Expression Genes

The replicate multivariate analysis of transcript splicing (rMATS, v3.0.8) was used to detect alternative splicing (AS) events [25]. The obtained alternative splicing events were divided into five types, including retained intron (RI), skipped exon (SE), alternative 3′ splice sites (A3SS), alternative 5′ splice sites (A5SS) and mutually exclusive exons (MXE). The differential AS events between the GMI1000 infected groups (6, 12, 24, 48 and 72 hpi) and the control group (0 hpi) were identified by changes of the inclusion level (ΔIncLevel). ΔIncLevel = average (IncLevel_infected_)—average (IncLevel_control_). IncLevel = (*I*/*li*)/(*S/ls* + *I/li*), where *I* represents the count of reads mapping the exon inclusion isoform, *S* represents the count of reads mapping the exon skipping isoform, *Ii* represents the effective length of the exon inclusion isoform, *ls* represents the effective length of the exon skipping isoform. Detailed descriptions about IncLevel algorithm can be found in the supporting information by Shen et.al. [25]. The AS events with |ΔIncLevel| > 0.05 and padj < 0.05 were identified as differential alternative splicing events (DSEs). DESeq R package (1.18.0) was used to perform the differential expression analysis. Genes with adjusted *p*-values < 0.05 and expression fold changes > 2 were determined as differentially expressed genes.

### 2.5. Function Annotation of RNA Sequence Data

The GOseq R package was used to carry out a gene ontology (GO) enrichment analysis of differential alternative splicing genes (DSGs) and expressed genes (DEGs) [26]. The significantly enriched GO terms were determined at an adjusted *p*-value < 0.05. KOBAS software was used to test the statistical enrichment of DSGs in the KEGG pathways [27]. The enriched pathways are shown in the figures based on statistical significance (adjusted *p*-values < 0.05).

### 2.6. Splicing Factors/RNA Bindng Poteins(SF/RBPs) Analysis

All the SF/RBPs genes were collected from dataset derived from Calixto et al., which was deposited in the DRYAD repository (http://dx.doi.org/10.5061/dryad.fk1cj47) [16].

### 2.7. RT-PCR Validation

A volume of 10 µL was used to perform the RT-PCR reactions. Primer pairs designed for each gene were based on selected AS isoforms using Primer 5.0. ATSKP1(AT1G75950) was used as an internal reference for normalization. The primer pairs for RT-PCR analysis are displayed in Table S2. The PCR cycling conditions were performed as follows: 1 cycle of 94 °C for 5 min, followed by variable cycles (25 to 32) of 95 °C for 30 s, 55 °C for 30 s, 72 °C for 2 min, and an extension of 3 min at 72 °C. The PCR products were run and visualized in 1–3% agarose gel under UV light.

## 3. Results

### 3.1. Identification of DSEs in Arabidopsis Root after GMI1000 Infection

**Figure 1:**
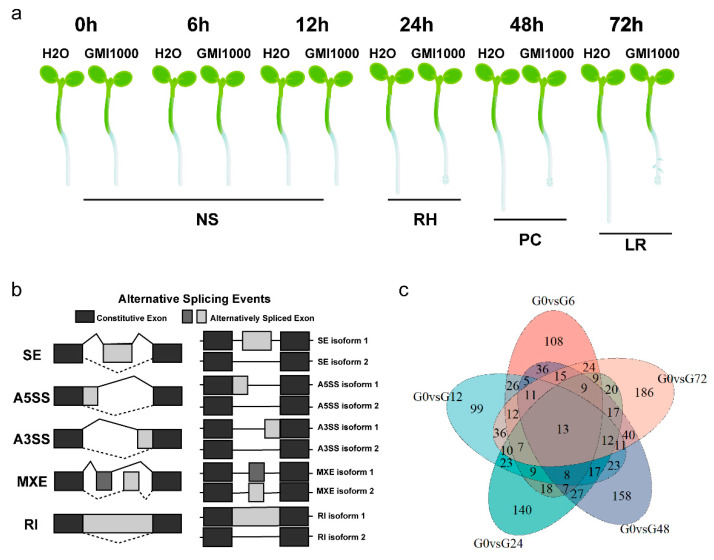
Differential alternative splicing events identified in *Arabidopsis thaliana* roots challenged with *Ralstonia* *solanacearum*. (**a**) Model of root symptoms following GMI1000 infection in our previous study. NS = no symptom, RH = root hair emergence, PC = primary root growth arrest and cell death and LR = lateral root emergence. (**b**) Each type of alternative splicing (AS) events detected by rMATs in our RNA-seq data: skipped exon (SE), alternative 5′ splice site (A5SS), alternative 3′ splice site (A3SS), mutually exclusive exons (MXE) and retained introns (RI). Each type of AS event produces two isoforms, namely isoform 1 and isoform 2, which are identified and qualified by junction reads only. (**c**) Comparison of differential alternative splicing genes (DSGs) at different time points.

In our previous study, changes in root morphology over time following infection by *R. solanacearum* were divided into the following periods: no symptoms (NS) from 0 to 12 hpi, root hair emergence (RH) at 12 to 24 hpi, primary root growth arrest and cell death (PC) at 24 to 48 hpi and lateral root emergence (LR) at 48 to 72 hpi (Figure 1a) [9]. To understand the transcriptional reprogramming that occurs in the root during the early stages of infection with *R. solanacearum*, RNA-seq analysis was performed on seven-day-old Arabidopsis seedlings at 0, 6, 12, 24, 48 and 72 h after infection of roots by GMI1000, with three biological replicates per time point. Thousands of DEGs were involved in diverse functions at the early stages of infection. In this study, we investigated the AS changes that occurred in the seedlings by mining our previously published RNA-seq data. In order to identify the specific AS events that were responsive to GMI1000 infection, we compared the AS events between G0 (GMI1000-infected roots at 0 h) and those at five time points after infection (G6, G12, G24, G48 and G72). The AS events with |ΔIncLevel| > 0.05 and padj < 0.05 were identified as DSEs. A total of 1491 DSEs were revealed, including 909 retained introns (RI), 268 alternative 3′ splice sites (A3SS), 165 alternative 5′ splice sites (A5SS), 148 skipped exons (SE) and 1 mutually exclusive exon (MXE) (Figure 1b and Table S3). The percentage occurrence of these five types of DSE indicated that RI was the most abundant, followed by A3SS, A5SS, SE and MXE. Genes undergoing DSEs were defined as differential AS genes (DSGs). In total, 1135 DSGs were identified. Of these, ~60% occurred transiently at a unique time point, and ~40% appeared at two or more time points. Only 13 DSGs lasted over the whole time course (Figure 1c and Table S4).

### 3.2. Comparison of AS and Transcriptional Regulation in Response to R. solanacearum Infection

**Figure 2:**
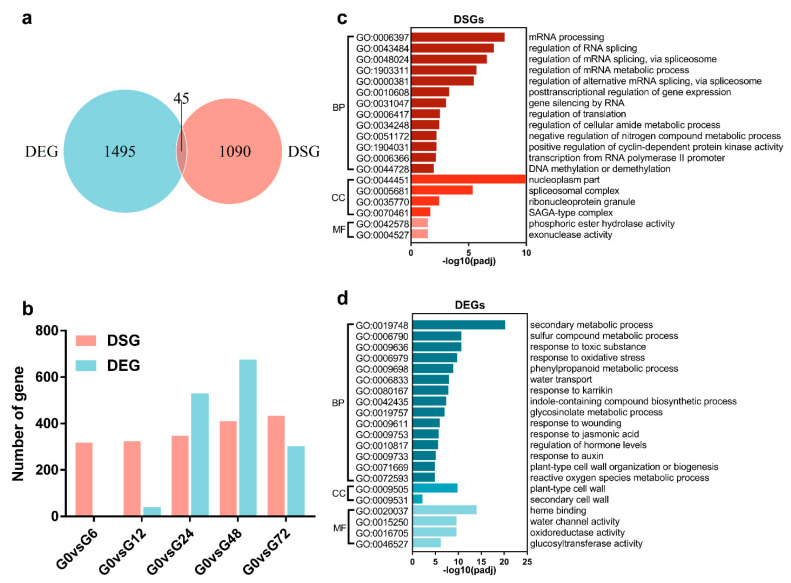
Comparison analysis of differentially spliced genes (DSGs) and differentially expressed genes (DEGs) in response to *R. solanacearum* infection. (**a**) Range comparison for DSGs and DEGs. (**b**) Number comparison for DSGs and DEGs over the time course. (**c**,**d**) Functional enrichment analysis of DSGs and DEGs. GO: gene ontology. BP: biological process. CC: cellular component. MF: molecular function.

Using stringent criteria of at least a two-fold change in expression and a padj < 0.05, 1540 genes were identified as DEGs between G0 and five time points after infection (G6, G12, G24, G48 and G72) (Table S5). To understand the relationship between AS and transcriptional regulation, we compared the DSGs and DEGs in response to *R. solanacearum* infection. A Venn diagram showed that only 45 genes were common between the DEG and DSG sets, while the remaining 1495 DEGs and 1090 DSGs were uniquely regulated at the transcriptional and AS levels (Figure 2a and Table S6). This indicated that AS may not directly regulate DEGs in most cases during Arabidopsis root defense against *R. solanacearum*. Subsequently, the dynamics of the changes in DEGs and DSGs were compared by plotting a line chart (Figure 2b). Interestingly, 318 of the DSGs and only one of the DEGs were detected within six h of *R. solanacearum* infection (Figure 2b), which indicated that the AS events took place much more rapidly in response to the attack by *R. solanacearum*. During the whole infection process, the number of DSGs increased slowly, but the DEG count significantly increased at 24 hpi and peaked at 48 hpi, before decreasing at 72 hpi (Figure 2b). A gene ontology (GO) enrichment analysis was performed to identify the functions of the DSGs and DEGs (Figure 2c,d). Over-representation of the GO categories for the DSGs were related mainly to “RNA processing”, “regulation of RNA splicing”, “gene silencing by RNA” and “regulation of translation” (Figure 2c). Whereas the enriched GO terms for the DEGs included “secondary metabolic process”, “response to oxidative stress”, “regulation of hormones” and “plant cell wall organization or biogenesis” (Figure 2d). These results suggested that the regulation of mRNA stability and post-translation may be another strategy used to control plant defense against *R. solanacearum*. Moreover, the epigenetic process-related GO term “DNA methylation or demethylation” was over-represented in the DSGs (Figure 2c). Given that DNA methylation was found to be involved in splicing regulations through differential methylation frequencies in introns and exons [28,29], AS may turn gene transcriptions on or off by dynamically changing DNA modifications. Collectively, our data suggest that AS, along with transcription, modulates the plant defense against *R. solanacearum* infection.

### 3.3. Analysis of DSEs in Arabidopsis Root during GMI1000 Infection

**Figure 3:**
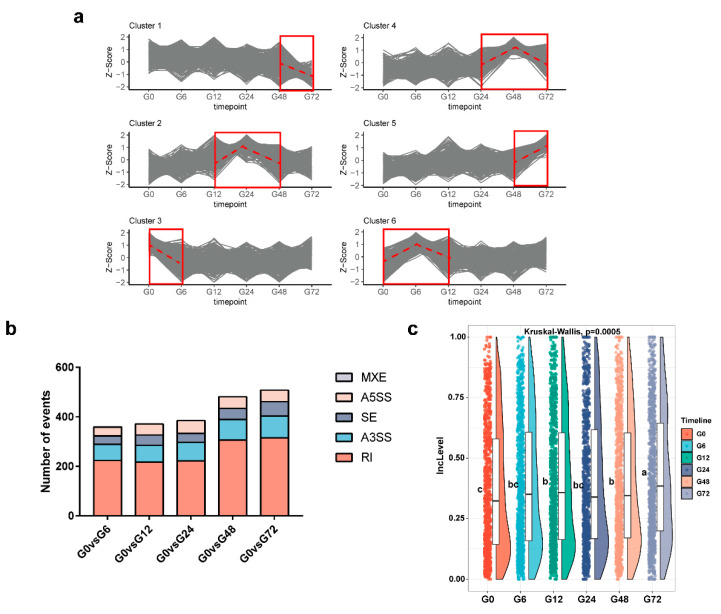
Analysis of differential alternative splicing events (DSEs). (**a**) Clustering analysis of DSEs according to standardized IncLevels (Z-scores). Red boxes and dotted lines show dynamic changes of Z-scores in six clusters. (**b**) Numbers of DSEs identified over the time course. (**c**) General IncLevel value changes of differential alternative splicing RI events in time order. Kruskal-Wallis method was used to represent the levels of significance. Dunn’s test was used for multiple comparisons. The “a”, “b”, “c” and “d” represent significant levels.

To examine the dynamic changes in DSEs in Arabidopsis roots in response to GMI1000 infection, a clustering analysis of IncLevel changes for all DSEs was performed using k-means over the time course of the infection. The DSEs were divided into six clusters according to their AS change profiles (Figure 3a). The IncLevels of Cluster 1 and Cluster 5, a total of 529 AS events, did not show significant changes until 72 hpi, suggesting that these AS events may be involved later in the infection process. Cluster 2 (216 AS events), which probably functions at the RH stage, increased at 12 hpi, peaked at 24 hpi and returned to the basal level at 48 hpi. Cluster 4 (245 AS events) showed a peak at 48 hpi (Figure 3a), which may have led to root growth inhibition and cell death at the root tip. Interestingly, Cluster 3 and Cluster 6, consisting of 501 AS events, exhibited rapid AS changes at six hpi (Figure 3a), which perhaps was related to the entry of *R. solanacearum* into the endodermis. These specific AS changes at different time points may reflect the complex process of *R. solanacearum* infection, which involves crossing several layers of root cells. Moreover, the number of DSEs steadily increased following infection by the pathogen and peaked at 72 h after the onset of infection (Figure 3b), suggesting that Arabidopsis produced more and more DSEs over time in response to the presence of GMI1000. Intriguingly, the number of DSEs suddenly increased by over 25% at 48 hpi, an event which may have been related to the emergence of symptoms, including cell death and the development of lateral roots. It has been reported that RIs are the most prevalent type of AS in Arabidopsis. In our study, the IncLevels of 909 RI events showed significant changes during GMI1000 infection; some RI IncLevel values increased, some decreased and others exhibited irregular changes (Table S7). We analyzed all of the RI IncLevel values during GMI1000 infection and found that they exhibited steady increases throughout the infection period (Figure 3c), indicating that the RI transcripts were enhanced by pathogen infection. Taken together, GMI1000 infection resulted in dynamic AS changes in hundreds of genes and an increased frequency of RIs.

### 3.4. Splicing Factors and RNA-Binding Proteins are Regulated at AS and Transcriptional Levels Following R. solanacearum Infection

**Figure 4:**
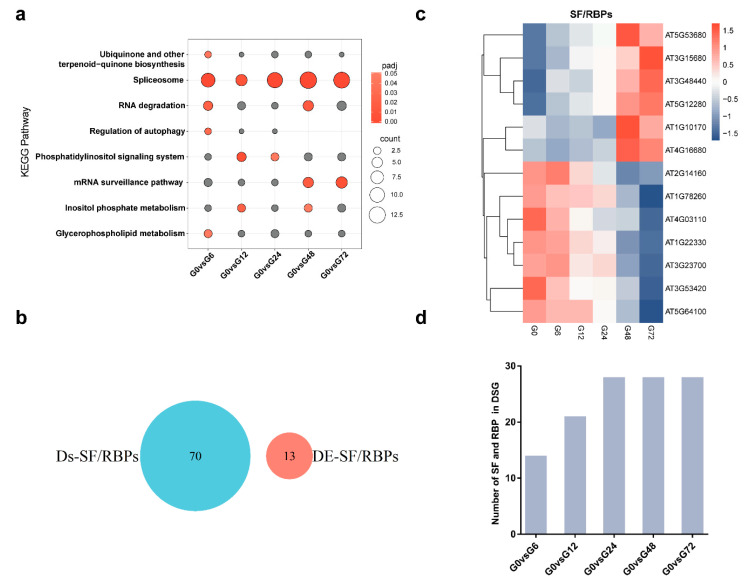
Analysis of differentially alternative splicing and differential expression splicing factor/RNA binding protein (SF/RBP) genes in response to *R. solanacearum* infection. (**a**) KEGG pathway enrichment for DSG at the indicated time points revealed that the “spliceosome” pathway was the most enriched. The colors of the bubbles indicate the enrichment significance. Gray represents padj > 0.05, while red represents padj < 0.05. The number of enriched genes is indicated by the scale of the bubbles. (**b**) Range comparison for differentially spliced SF-RBPs genes (DS-SF/RBPs) and differentially expression SF/RBPs genes (DE-SF/RBPs). (**c**) Clustering analysis of DE-SF/RBP expression patterns in chronological order. (**d**) Number of DS-SF/RBPs in Arabidopsis roots infected with *R. solanacearum* in the time course.

Using GO, we determined that terms associated with the “spliceosome” pathway were the most significantly enriched at each time point in the infection process (Figure 4a), suggesting that *R. solanacearum* infection resulted in AS. Splicing factors and RBPs are key regulators, responsible for spliceosome assembly, splice site recognition and intron removal in plants [20]. Thus, we investigated the changes in SF/RBPs at the AS and transcriptional levels following *R. solanacearum* infection, according to the SF/RBPs reference gene lists [16]. Thirteen and 70 SF/RBP genes were identified as DEGs and DSGs, respectively (Table S8). Notably, no SF/RBP genes overlapped between the differentially expressed (DE) and differentially spliced (DS) SF/RBP genes (Figure 4b and Table S8). These results suggested that different SF/RBPs are subjected to AS or transcriptional regulation separately, following *R. solanacearum* infection. Interestingly, seven SF/RBP DEGs displayed significant decreases in gene expressions due to *R. solanacearum* infection, while the remainder were upregulated (Figure 4c), implying that the SF/RBP DEGs are specific to *R. solanacearum* pathogenesis. With regard to the DS SF/RBP genes, 14 were rapidly detected at six hpi. Following infection, the number of AS SF/RBPs in the Arabidopsis root increased to a maximum at 24 hpi and then remained stable until 72 hpi (Figure 4d). However, the list of AS SF/RBP genes changed at different time points after *R. solanacearum* treatment (Figure S1), suggesting that the AS of various SF/RBPs is infection stage-specific. This is different from the strain specificity seen in SF/RBP DSGs. *UBA2A* and *UBA2C*, the SF/RBP genes involved in wound-induced responses [30], were detected among our DSGs. Furthermore, we identified two isoforms of *PAT1* in our data. RNA-binding protein PAT1 is localized in processing bodies and regulates mRNA decay. Mutations of *PAT1* derepress immune reactions that are dependent on the immunity receptor SUMM2 [31]. The isoforms of *PAT1* seen in our data harbored a different 5′ untranslated region that may suppress plant immunity by increasing the ratio of RI transcripts. Taken together, both the AS and transcriptional regulation in SF/RBPs contribute to changes in the transcriptome and affect plant defenses against *R. solanacearum* in different ways.

### 3.5. Identification of Various Defense-Related Genes Subjected to AS Modulation Following R. solanacearum Infection

**Figure 5:**
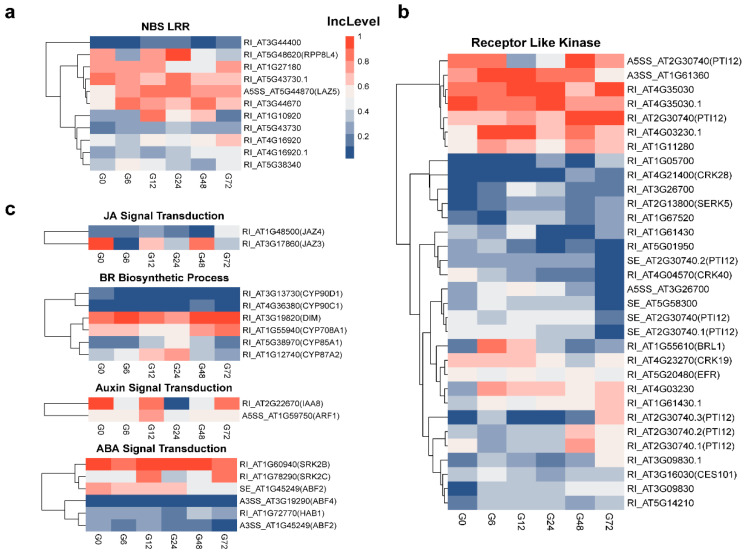
Clustering analysis of defense-related gene IncLevel value patterns. (**a**–**c**) Three groups of defense-related genes identified in DSGs, namely NBS-LRR (R genes) (**a**), receptor-like kinase (RLK) **(b**) and plant hormone signaling or biosynthesis genes (**c**). In the heatmap, red means a high IncLevel, while blue means a low IncLevel. Event names on the right are composed of the AS type and gene locus. Different isoforms for the same gene are noted by “.1”,”.2” and so forth.

Cell-surface immune receptor PRRs and intracellular receptor NBS-LRRs play very critical roles in activating plant immunity [3]. To test whether these receptors are subject to AS-based regulation during plant root defense against *R. solanacearum*, we carefully looked through 1135 DSGs and found that the IncLevel values of nine nucleotide binding domain and leucine-rich repeat (NB-LRR) protein genes changed following infection (Figure 5a). *LOV1* encodes a coiled-coil NB-LRR protein. Loss of *LOV1* function reduces the susceptibility to victorin, a compound from the fungus *Cochliobolus victoriae* and enhances plant resistance to the fungus [32]. Two *LOV1* transcript isoforms identified in our data differed due to the removal or retainment of intron 1 (Figure S2), which alters the N-terminal sequence of the predicted protein. The AS isoform 1 of *LOV1* increased at 12 hpi and decreased at 72 hpi (Figure 5a and Figure S2), suggesting that it may have a special function in the RH and PC stages of *R. solanacearum* infection. LAZ5, an RPS4-like R protein, triggers hypersensitive cell death [33]. Changes in the AS of *LAZ5* were rapidly induced at six hpi and peaked at 24 hpi (Figure 5a and Figure S2), which may have been associated with the occurrence of cell death at 48 hpi. Moreover, 22 RLKs, including the EF-Tu receptor (EFR), were found to be differentially spliced after infection with *R. solanacearum* (Figure 5b). EFR functions as the receptor for bacterial PAMP EF-Tu and triggers the PTI to defend against the phytopathogen [34]. EFR-overexpressing plants exhibit an enhanced resistance to *R. solanacearum* [35,36]. As plant hormone signals are important to the plant defense response against *R. solanacearum*, we investigated the dynamic AS changes in phytohormone-associated genes in response to this pathogen and found 15 DSGs associated with the auxin, jasmonic acid and abscisic acid signaling pathways and brassinosteroid biosynthesis (Figure 5c). Jasmonate ZIM domain genes (*JAZ3* and *JAZ4*), key negative regulators of the jasmonic acid signaling pathway, showed differential AS patterns at 72 hpi (Figure 5c). It has been reported that *JAZ4* enhances the Arabidopsis resistance to the hemi-biotrophic pathogen *P. syringae* [37]. Three of eight brassinosteroid biosynthesis genes (*CYP90D1, CYP90C1* and *CYP85A1*) exhibited differential AS patterns during infection. Moreover, five differential splicing genes (*HAB1, SRK2C, SRK2B*, *ABF4* and *ABF2*) were associated with abscisic acid signal transduction. Therefore, our results suggested that AS is an important further layer of regulation that controls genes that are responsive to pathogen attacks.

**Figure 6:**
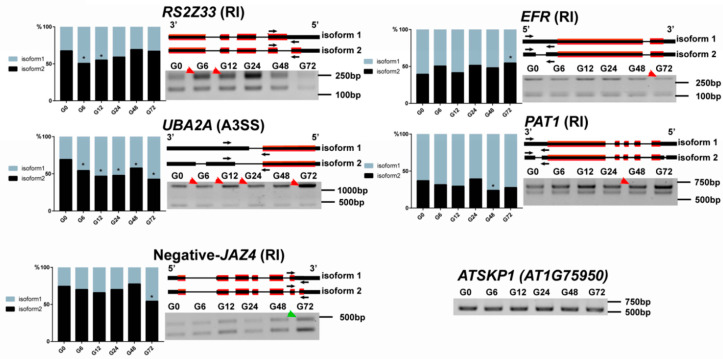
Experimental validation of defense-related events by reverse transcription (RT)-PCR in *Arabidopsis thaliana*. The bar charts show the relative percentages of isoform1 and isoform 2 during *R. solanacearum* infection according to IncLevel values. The asterisks show the significance compared to 0 h (G0). For the transcripts model, the red lines represent the coding areas and black arrows represent the RT-PCR primer binding sites. *ATSKP1*(*AT1G75950*) was used as an internal reference for normalization. Red arrows in the RT-PCR results show the consistency between the results and bar plots, while the green arrow shows the nonconsistency.

To confirm the occurrence of AS events in the DSGs identified by RNA-seq, we designed specific primers for several genes selected from the SF/RBPs, PRRs and phytohormone signaling components and performed reverse transcription (RT)-PCR to validate the predicted AS patterns during GMI1000 infection. Several genes could not be detected, maybe due to their excessively low expression levels. Four detected DSGs were consistent with the AS patterns predicted by the RNA-seq data (Figure 6). For example, the RI isoform of *RS2Z33* increased obviously at 6 h and 12 h after GMI1000 infection, which is in-line with the DNA bands’ gray value change. The RI isoform of *EFR* decreased 72 h after GMI1000 infection, consistent with the change in the RT-PCR results. These results further confirm the accuracy of our bioinformatics analysis. As expected, there were some RT-PCR results unable to consist with the RNA-Seq data, like *JAZ4* (Figure 6).

## 4. Discussion

Plants are subverted by a wide range of pathogens, including fungi, viruses and bacteria, during their growth and development processes. Therefore, plants have evolved complicated molecular mechanisms to respond to these stresses. Current knowledge about plant defenses against pathogens mainly focuses on immune signal transduction and transcriptional changes. Little is known about post-transcriptional regulation in plant defenses. Alternative splicing is one of the most important post-transcriptional regulatory mechanisms and plays a critical role in plant development. In this study, we analyzed the features of the genome-wide AS landscape in Arabidopsis roots infected with *R. solanacearum* and revealed a dynamic global profiling of the AS landscape in response to *R. solanacearum*, suggesting that this pathogen interferes with the host AS regulatory mechanism and affects the Arabidopsis root defense.

A total of 1491 AS events, including RI, SE, A3SS, A5SS and MXE, were identified as DSEs during the Arabidopsis root-*R. solanacearum* interaction, and the isoforms of 1135 genes in Arabidopsis root changed in response to the infection (Figure 2a). These were categorized into GO terms for mRNA processing and metabolism, epigenetic processing, etc. (Figure 2c). We also found 1540 genes subjected to transcriptional regulation that were enriched in the GO terms correlated with the secondary metabolic process and response to biotic stress (Figure 2d). Very few genes (only 45) overlapped between the DEGs and DSGs in response to *R. solanacearum* infection (Figure 2a). Consistent with our data, few overlapped genes between DSGs and DEGs were found previously in Nicotiana attenuata challenged with insect herbivory and wheat treated with strip rust fungus [38,39]. Furthermore, these two studies showed that the DEGs and DSGs were enriched in different GO terms, which concurred with our data. Compared with the DSGs enriched in the GO terms “RNA processing and metabolism” and “DNA methylation and demethylation” found in our studies, those regulated in response to insect attacks are over-represented in GO terms such as “nucleotide kinase activity”, “exopeptidase activity” and “cellular biogenic amine biosynthetic process” [38], while DSGs involved in wheat-strip rust interactions are categorized into the terms “transport”, “peroxisome”, “lncRNA metabolism” and “protein modification” [39]. These differences may be related to the pathogen lifestyle, but we cannot rule out the possibility that the AS regulation mechanism is also plant species-specific.

Rapid and dynamic changes of AS events occurred in Arabidopsis roots during *R. solanacearum* infection (Figure 3b). In-line with this, AS events in Arabidopsis were previously detected within 40 to 60 min of the cooling treatment [16]. In mammals, when neurons are stimulated and intracellular sterols change rapidly, AS is activated within several minutes [40,41]. Compared with the rapid changes in AS that occurred in response to *R. solanacearum*, gene expression responses lagged behind at around 18 h after infection (Figure 2b). A microarray analysis of the leaf transcriptome shows that very few genes are differentially expressed in the 6- and 12-h post-*R. solanacearum* inoculation, which is a similar finding to that obtained from our root transcriptome data [9,24]. Thus, changes in AS occur more quickly than changes in the level of transcription, indicating that AS may be one of the earliest responses to *R. solanacearum* infection. It is proposed that AS provides a means to fine-tune the abundance of transcripts by regulating functional and nonfunctional transcripts without de novo transcription or protein production, which could be a more cost-effective way to fit the condition. Some genes displayed transient changes in AS patterns at unique time points (Figure 3a). Therefore, we hypothesized that those AS changes may contribute to pathogen perceptions, initial immune responses and physiological processes in response to the pathogen crossing and multiplying in different plant tissues. However, whether these changes in AS patterns are beneficial or harmful to Arabidopsis remains unclear. Interestingly, about 61% of all DSEs involved RI, which was the predominant type of AS event. The IncLevels of RI events increased steadily during the *R. solanacearum* infection (Figure 3c). Most of the RI transcripts were predicted to generate a premature stop codon that would lead to the production of nonfunctional mRNAs [11]. Given that GMI1000 is a virulent strain on Arabidopsis, it is reasonable to hypothesize that the increasing DSEs and RI transcripts have a negative effect on the Arabidopsis defense during *R. solanacearum* infection.

We found that the AS patterns of 70 SF/RBP genes exhibited many changes, while only a few SF/RBPs were differentially expressed in response to *R. solanacearum* (Figure 4b). These results reflect that SF/RBPs are mainly regulated at the AS level and not at the transcriptional level. Some of the SFs regulated by AS in this study are involved in plant responses to biotic stresses. For example, the SFs SCL33, SR45a, RS40, RS41, U2AF65A and RZ1C have shown AS changes under PAMP treatment [42]. The outcome of AS is mostly determined by the abundance, activity and localization of SF/RBPs, which recruit the spliceosome for intron removal [22,23]. We presume that any alterations to the SF/RBPs may change the AS patterns of genes and affect plant defenses to *R. solanacearum*. This hypothesis is supported by the role of SF/RBPs in plant-pathogen interactions. For example, the RLKs *SNC4* and *CERK1* were shown to undergo AS in response to PAMPs [43]. Moreover, SUA and RSN2, two SFs, are required for the proper splicing of *SNC4* and *CERK1* pre-mRNAs. Mutations in SUA and RSN2 suppress plant defenses activated by chitin [43]. Loss of function of the splicing regulator SR45 results in differential AS patterns and an increased resistance to the bacterial pathogen *P. syringae* PmaDG3 [44]. In addition, effectors from pathogens inhibit RBP function and enhance plant sensitivity to the *P. syringae* effector HopU1, which targets glycine-rich RBP GRP7, reduces the ability of GPR7 to bind to the PAMP receptors EFR and FLS2 and suppresses plant immunity [45]. Further experimental investigations are expected to draw on the biological relevance of SF/RBPs in plant defenses to *R. solanacearum*.

During the arm race between plants and phytopathogens, plants have acquired PTI and ETI to defend against their attackers. The membrane receptor RLKs and RLPs recognize PAMPs from microbes and trigger PTI [3]. Whereas intracellular NBS-LRR proteins directly or indirectly sense effectors secreted by the pathogen into the host cell and activate ETI. In this study, 22 RLKs showed DS patterns after *R. solanacearum* infection. EFRs, the receptors for PAMP EF-Tu, were found to have two isoforms, and the IncLevel value decreased after *R. solanacearum* infection (Figure 5b). Tobacco *Nt-Sd-RLK* has two isoforms, which play different roles in the recognition of lipopolysaccharides [46]. Additionally, the abnormal AS of chitin receptor CERK1 increases the plant susceptibility to *P. syringae* [42], and the expression of *AtEFR* in Nicotiana benthamiana and tomatoes results in an enhanced resistance to *R. solanacearum* [36]. It would be of interest to know what roles isoform 2 of *EFR* has in plant defenses against to *R. solanacearum*. We also found the IncLevel values of nine NBS-LRR genes significantly changed in Arabidopsis roots after the *R. solanacearum* treatment (Figure 5a). This phenomenon has also been identified in tobacco, tomatoes, potatoes, rice and barley [21]. In Arabidopsis, the abundance of alternatively spliced isoforms of the R protein RPS4 shows dynamic regulation in response to the effector AvrRps4, and the ratio between the AS isoforms affects RPS4-mediated ETI [47]. It is possible that the ratio changes in the AS isoforms of these nine NBS-LRR genes perturb ETI and facilitate *R. solanacearum* invasion.

Phytohormones are also well-known to be critical for plant resistance to various pathogens. It has been reported that AS is a regulator of specific steps in jasmonic acid, brassinosteroid and salicylic acid signaling [13]. In this study, the differential AS isoforms of five abscisic acid signaling genes (*HAB1, SRK2C, SRK2B, ABF4* and *ABF2*) have been identified (Figure 5c). Interestingly, the disruption of ABA receptors enhances the susceptibility to *R. solanacearum* [9]. Furthermore, two ABA-insensitive mutants, *abi1-1* and *abi2-1*, also show an increased sensitivity to *R. solanacearum* [48]. Therefore, we guess the AS isoforms of these five ABA signaling-related genes may interfere with ABA signaling and promote *R. solanacearum* infection.

Taken together, the results indicated that AS acts as a new and conserved mechanism to modulate plant-microbe interactions. However, we cannot distinguish whether the AS events identified were resulted from transcriptional influence or decay, for only steady-state RNA can be measured through RNA-seq. Considering the migration of *R. solanacearum* to leaves, we only analyzed AS in the Arabidopsis seeding roots in response to the *R. solanacearum* infection. Thus, a number of AS genes could be overlooked in our study in the leaves during the late stage of infection.

## 5. Conclusions

Both transcriptional and AS regulations are critical for plant defense. In this study, AS regulation made much faster responses than gene expression in response to *R. solanacearum*. DSGs were enriched into GO terms related with post-transcriptional regulations such as “RNA processing and metabolism” and the “regulation of translation”. Besides less common genes between DSGs and DEGs, the AS and transcriptional mechanisms regulate completely different SF/RBPs following *R. solanacearum* infection. Our results suggested AS regulation may act as an independent layer in plant defenses against *R. solanacearum*. We also identified AS events and splice isoforms in defense-related genes, greatly expanding the AS gene repertoire involved in plant defense and introducing AS as a novel process in their regulation. Our study provides new insights into understanding Arabidopsis dynamic transcriptome reprogramming during *Ralstonia solanacearum* infection.

## Supplementary Materials

The following are available online at https://www.mdpi.com/2073-4425/11/9/1078/s1. Figure S1: Comparison of differentially alternative splicing SF/RBPs (DS-SF/RBPs) in different time points, Figure S2: AS isoforms model and isoform proportion changes of LOV1 and LAZ5, Table S1: The clean reads ratio and mapped reads ratio against the Arabidopsis reference, Table S2: Primer sequences of candidate genes in Arabidopsis for RT-PCR. Table S3: Differential alternative splicing events in Arabidopsis roots after *Ralstonia solanacearum* infection, Table S4: Differential alternative splicing genes in Arabidopsis roots after *Ralstonia solanacearum* infection, Table S5: List of differentially expressed genes identified upon *Ralstonia solanacearum* infection, Table S6: Differential alternative splicing and expressed genes upon *Ralstonia solanacearum* infection, Table S7: Distribution of IncLevel changes among RI events after *Ralstonia solanacearum* infection, Table S8: Differential expressed SF/RBPs (DE-SF/RBPs) and differential spliced SF/RBPs (DS-SF/RBPs) upon *Ralstonia solanacearum* infection.
