# Supplementary material for: Global Profiling of Dynamic Alternative Splicing Modulation in Arabidopsis Root upon Ralstonia solanacearum Infection
Source: Genes (Basel). 2020 Sep 15;11(9):1078. doi: 10.3390/genes11091078 (PMC7563316; doi:10.3390/genes11091078)
Supplement: Supplementary file 1 [file genes-11-01078-s001.zip › FigureS1-S2.docx]

| 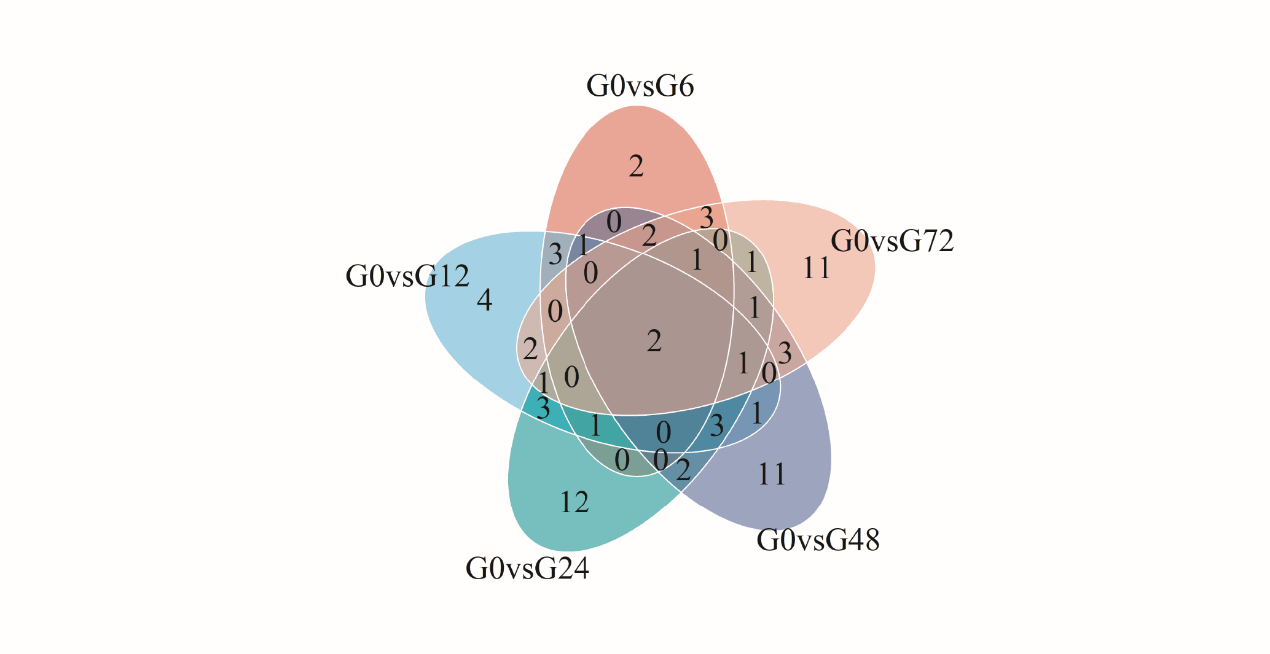 |
| --- |

**Figure S1**. Comparison of differentially alternative splicing SF/RBPs (DS-SF/RBPs) in different time points.

| 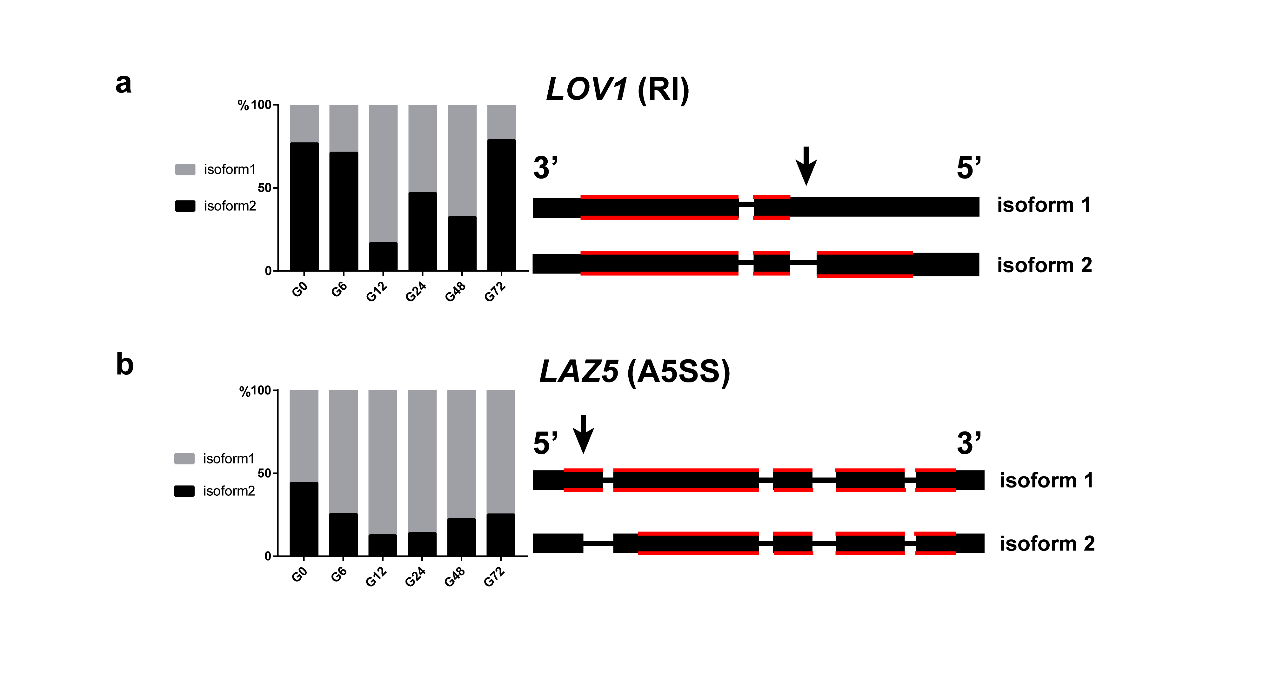 |
| --- |

**Figure S2.** AS isoforms model and isoform proportion change of LOV1 (**a**) and LAZ5(**b**). The bar charts show relative percentage of isoform1 and isoform2 during *R. solanacearum* infection. AS types are noted in brackets behind gene names. For isoforms model, area with red lines represent coding area, and black arrows show where AS occurred.
